# A Hybrid Machine Learning Approach to Energy Consumption and Road Emissions Modeling of CNG Vehicles Based on Chassis Dynamometer Data and Road Load Power

**DOI:** 10.3390/ma19122503

**Published:** 2026-06-10

**Authors:** Artur Jaworski, Krzysztof Balawender, Hubert Kuszewski, Bożena Babiarz, Dariusz Szpica

**Affiliations:** 1 Faculty of Mechanical Engineering and Aeronautics, Rzeszow University of Technology, 12 Powstancow Warszawy Ave., 35-029 Rzeszow, Poland; kbalawen@prz.edu.pl (K.B.); hkuszews@prz.edu.pl (H.K.); 2 Faculty of Civil and Environmental Engineering and Architecture, Rzeszow University of Technology, 12 Powstancow Warszawy Ave., 35-029 Rzeszow, Poland; bbabiarz@prz.edu.pl; 3 Faculty of Mechanical Engineering, Bialystok University of Technology, 45C Wiejska Str., 15-351 Bialystok, Poland; d.szpica@pb.edu.pl

**Keywords:** compressed natural gas (CNG) vehicle emissions, road load power, emission modeling and prediction, energy consumption, chassis dynamometer testing, real-world driving emissions (RDE)

## Abstract

**Highlights:**

- Covers energy consumption and multiple gaseous pollutants (CO_2_, CO, HC, NO_X_, CH_4_), not only CO_2_ as in most studies.
- Introduces road load power as an additional predictor beyond speed and acceleration.
- Trained on precise lab data and validated in two urban RDE tests with different driving dynamics.
- Enables CH_4_ estimation in RDE despite limited PEMS capability using lab-based ML modelling.

**Abstract:**

This study presents a comparative analysis of energy consumption and gaseous emissions from a compressed natural gas (CNG)-fueled vehicle under real driving emissions (RDE) conditions and values predicted using machine learning (ML) models developed from chassis dynamometer data. The analyzed components included energy consumption (EC) as well as carbon dioxide (CO_2_), carbon monoxide (CO), total hydrocarbons (HC), methane (CH_4_), and nitrogen oxides (NO_X_). The models were trained using a limited set of easily accessible predictors, namely vehicle speed and acceleration. A hybrid modelling approach was proposed, combining laboratory data with validation under real-world conditions. Additionally, road load power (*P_rl_*) was introduced as a novel predictor representing vehicle operating load. The results demonstrate that the models effectively capture emission trends, with the highest agreement obtained for CO, CO_2_. The inclusion of *P_rl_* improved prediction accuracy, which increased from approximately 64% to 71% for CO and from 57% to 61% for HC. For CO_2_, the model achieved about 80–82% agreement with RDE measurements, with analogous levels obtained for EC. A key advantage of the proposed methodology is its reliance on a limited number of input variables, which enhances practical applicability while maintaining satisfactory accuracy. Furthermore, the use of precise laboratory data improves model robustness, and the approach enables the estimation of methane (CH_4_), which is typically not measured by standard portable emissions measurement systems (PEMSs). The results confirm the effectiveness of the hybrid ML framework and highlight the importance of incorporating load-related parameters in real-world emissions and energy consumption modeling.

## 1. Introduction

In recent years, increasing attention has been paid to reducing energy consumption (EC) and pollutant emissions from road transport, which is a major source of greenhouse gases and compounds harmful to human health [1,2]. In response to increasingly stringent emission regulations and the need to mitigate the environmental impact of transport, intensive research efforts have focused on the assessment and prediction of pollutant emissions under real-world vehicle operating conditions [3,4]. Studies conducted under on-road conditions are particularly important because they account for actual vehicle operation and traffic variability that cannot be fully reproduced in laboratory testing [5,6,7,8].

In practice, pollutant emissions under real-world driving conditions are commonly measured using portable emissions measurement systems (PEMS), which enable direct on-road emission measurements [9,10]. At the same time, regression-based emission models are increasingly applied to estimate emissions based on parameters describing vehicle motion and operating conditions [11,12]. In many studies, these models rely mainly on vehicle speed and acceleration, which are easily available from on-board diagnostics (OBD) systems or vehicle data loggers [13,14,15]. Although this approach enables relatively simple emission modeling, it does not always adequately represent the actual load of the vehicle powertrain [16].

Under real-world conditions, propulsion power demand and pollutant emissions are also influenced by road grade, rolling resistance, and aerodynamic drag [17,18,19]. However, accurate determination of these resistances under on-road conditions is difficult, which limits their use in emission models. In contrast, chassis dynamometer testing enables controlled reproduction of driving resistances and precise measurement of operating parameters [20,21,22]. This creates the possibility of developing EC and emission models based on laboratory data while accounting for actual vehicle load conditions.

The importance of alternative fuels, particularly compressed natural gas (CNG), is also increasing in the context of transport decarbonization. Compared to conventional fuels, CNG can reduce emissions of carbon dioxide (CO_2_), nitrogen oxides (NO_X_), and particulate matter, although methane (CH_4_) emissions remain an important issue because of their greenhouse impact. Consequently, increasing attention is being paid to emissions from CNG-fueled vehicles and to methods for their prediction under different operating conditions. Research on CNG-powered vehicles mainly concerns two areas: experimental evaluation of EC and emissions under laboratory and on-road conditions, and the development of models for estimating fuel consumption and emissions based on operational parameters. Experimental studies indicate that natural gas can reduce emissions of selected pollutants, particularly CO_2_, carbon monoxide (CO), and particulate matter; however, the environmental benefits strongly depend on vehicle operating conditions and driving cycles. It is also emphasized that the environmental assessment of CNG-powered vehicles should include emissions such as CH_4_, ammonia (NH_3_), and nitrous oxide (N_2_O), which are not always included in standard evaluations [23,24,25].

Studies focused on light-duty vehicles have shown that comparing laboratory and on-road results is essential for assessing the real-world applicability of CNG. Lejda et al. [23] demonstrated that a passenger car adapted for natural gas fueling emitted lower CO_2_ and CO emissions under CNG operation than under gasoline fueling, although significant differences between laboratory and on-road results were observed, especially for CO_2_. Similar conclusions were reported by Vojtíšek-Lom et al. [25], who showed that CNG-powered light commercial vehicles were characterized by low NO_X_, NH_3_, and CH_4_ emissions, while the overall environmental assessment strongly depended on the driving cycle and urban driving share.

Comparable observations have been reported for heavy-duty vehicles and buses fueled with CNG. Gioria et al. [24] demonstrated that, besides regulated pollutants, emissions of CH_4_, NH_3_, and fine particles are also important and strongly dependent on ambient temperature, cold-start conditions, and driving cycle characteristics. Studies discussed by Zhao et al. [26] also showed that although CNG vehicles can reduce selected conventional pollutants under urban conditions, the assessment of their environmental impact requires consideration of unregulated emissions and real-world operating conditions [24,26].

Additional real-world studies further confirm the complexity of emission behavior in natural gas-fueled vehicles. Dimaratos et al. [27] showed that a bi-fuel gasoline/CNG vehicle emitted lower CO and particle number emissions under natural gas operation, although NO_X_ emissions increased. The study also demonstrated the strong influence of start-up conditions and driving characteristics on emissions. Similarly, Lv et al. [28], in studies on CNG, liquefied natural gas (LNG), and hybrid buses, showed that pollutant emissions strongly depend on vehicle speed and driving cycle characteristics, particularly under dynamic operating conditions. These findings confirm that traffic variability and vehicle load significantly affect emissions, complicating their representation using simplified models.

Methane emissions are particularly important in natural gas-fueled vehicles. Pan et al. [29] demonstrated that real-world CH_4_ emissions may be substantially higher than values estimated using simplified inventory assumptions. Neglecting methane can therefore lead to underestimation of the climate impact of natural gas-powered vehicles. This is especially important for intensively operated urban fleets. Consequently, methane should be treated as a separate emission component requiring dedicated prediction and validation methods, particularly because direct CH_4_ measurements under on-road conditions are often difficult or unavailable [24,29].

Another important research direction concerns the modeling of fuel consumption and emissions in natural gas-powered vehicles. Zhao et al. [26] applied a localized version of the international vehicle emissions (IVE) model to natural gas-powered taxis using parameters such as vehicle specific power (VSP) and engine stress. Their results showed that vehicle speed alone is insufficient for accurate emission modeling and that incorporating load-related parameters significantly improves the interpretation of emission behavior [26].

Similar conclusions were reported by Madhusudhanan et al. [30], who developed a fuel consumption model for a CNG/biomethane-powered truck based on real-world operational data. In contrast to simplified approaches based only on speed and acceleration, their model included longitudinal vehicle dynamics, engine maps, road elevation, and vehicle mass variability. The authors emphasized that many existing models neglect the effects of road grade, aerodynamic drag, rolling resistance, and vehicle mass, limiting their ability to reproduce actual energy demand and engine load [30].

Despite the increasing interest in CNG-powered vehicles, predictive emission models dedicated to this fuel remain relatively limited. Mądziel [31] proposed a CO_2_ emission model for a natural gas-powered vehicle based on on-road and chassis dynamometer measurements using machine learning techniques. The study indicated that emission models dedicated to CNG vehicles are still at an early stage of development and are usually based on a limited number of predictors, mainly vehicle speed and acceleration, without detailed consideration of actual powertrain load conditions [31].

Based on the cited studies, it can be concluded that current research on CNG vehicles mainly focuses on comparing laboratory and on-road emissions or on models based on operational parameters such as vehicle speed, acceleration, VSP indicators, or onboard signals. However, approaches integrating high-accuracy laboratory data with instantaneous emission prediction under real-world conditions remain relatively limited. In many models, actual vehicle load related to road grade, rolling resistance, and aerodynamic drag is represented only indirectly or in a simplified way, which may reduce prediction accuracy. In addition, although EC can be determined using the carbon balance method based on CO_2_, CO, and HC emissions, this approach is rarely integrated into machine learning-based frameworks and is usually limited to aggregated analyses rather than instantaneous predictions. Despite the importance of methane emissions, CH_4_ is also not explicitly modeled in many studies, mainly because standard PEMSs do not include methane analyzers. At the same time, research on conventional CNG remains relevant due to the growing importance of biomethane (bio-CNG). After upgrading and purification, bio-CNG can achieve physicochemical properties comparable to fossil natural gas and meet the same fuel quality standards. Consequently, from the perspective of combustion and engine operation, bio-CNG can be considered functionally equivalent to conventional CNG in many applications [32]. This implies that models developed for conventional CNG vehicles may also be applicable to bio-CNG-powered vehicles, particularly regarding energy consumption and methane-related emissions [33].

Consequently, there is a need to develop models for CNG-fueled vehicles that combine high-quality laboratory data with applicability under real-world conditions. Unlike previous studies based mainly on VSP indicators or simplified speed–acceleration relationships, the present study applies road load power (*P_rl_*) as a physically interpretable parameter representing actual powertrain load under both laboratory and real-world operating conditions. In addition, the proposed approach includes instantaneous CH_4_ emission modeling and carbon-balance-based energy consumption determination within a unified machine learning framework developed for CNG-powered vehicles. Such models should explicitly incorporate driving resistances as predictors and extend the analysis to methane emissions and energy consumption determined using the carbon balance method. Therefore, the objective of the present study was to develop and validate models based on chassis dynamometer tests and apply them under real driving emissions (RDE) conditions while providing a framework applicable to both conventional CNG and bio-CNG.

The present study intentionally focused on a simplified and practically applicable model structure based solely on vehicle speed, acceleration, and road load power. These parameters can be determined directly during vehicle operation without the need for advanced measurement equipment, which constitutes an important practical advantage of the proposed approach.

## 2. Materials and Methods

### 2.1. Chassis Dynamometer Testing

**Figure 1:**
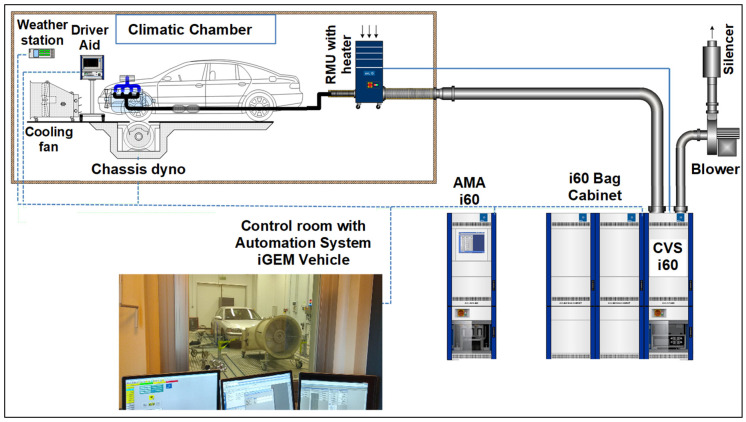
Schematic of the chassis dynamometer installed in a climatic chamber.

Laboratory experiments were conducted using a chassis dynamometer installed in a climatic chamber (Figure 1) at the Automotive Ecology Center of Rzeszow University of Technology. A detailed description of the test facility is provided in [34]. The measurement setup included, among others, an AVL CVS i60 (AVL List GmbH, Graz, Austria) exhaust dilution system and an AVL AMA i60 (AVL List GmbH, Graz, Austria) exhaust gas analyzer. The key specifications of these systems are summarized in Table 1.

In the present study, a CNG-fueled vehicle compliant with the Euro 3 emission standard was selected for experimental investigation. This choice was justified by its representativeness of a still-operating segment of the vehicle fleet, particularly in Central and Eastern European countries, as well as by its relevance for analyzing transitional technologies aimed at reducing exhaust emissions. In addition, the limited number of studies addressing CNG vehicles of this emission class indicates an existing research gap, which this study partially addresses. The main technical specifications of the tested vehicle, along with the road load simulated on the chassis dynamometer, are presented in Table 2. The road load function was determined using an alternative method based on vehicle data, in accordance with UN Regulation No. 154 [37]. According to this regulation, as an alternative to determining road load using coast-down testing or torque measurement methods, a default road load may be calculated based on vehicle parameters.

The default road load force *F_rl_* according to this method, is expressed by the following equation:

(1) $$F_{rl}=f_{0}+f_{1}\cdot V+f_{2}\cdot V^{2}$$

where

*f*_0_—the constant road load coefficient calculated using Equation (2), N [37]:

(2) $$f_{0}=0.140\cdot m_{t}$$

*f*_1_—the first-order road load coefficient is assumed to be equal to zero, N/(km/h);
*f*_2_—the second-order road load coefficient calculated using Equation (3), N/(km/h)^2^ [37]:

(3) $$f_{2}=\left(2.8\cdot 10^{-6}\cdot m_{t}\right)+\left(0.017\cdot W\cdot H\right)$$

*V*—vehicle speed, km/h;
*m_t_*—test vehicle mass, kg;
*W*—vehicle width, m;
*H*—vehicle height, m.

According to this methodology, the test vehicle mass *m_t_* is defined as the sum of the actual vehicle mass *m_R_*, 25 kg, and a representative mass corresponding to the vehicle load. The actual vehicle mass refers to the mass of the vehicle in running order, including the mass of any optional equipment installed in the given vehicle. The representative mass is expressed as the product of a coefficient *φ*, dependent on the vehicle category, and the maximum vehicle load. The test vehicle mass m_t_ is calculated using Equation (4):

(4) $$m_{t}=m_{R}+25+\varphi \cdot m_{vl}=m_{R}+25+\varphi \cdot \left(m_{l}-m_{ro}-m_{o}-25\right)$$

where

*m_R_*—actual vehicle mass, kg;
*m_vl_*—maximum vehicle load, kg;
*φ*—the load coefficient dependent on the vehicle category (*φ* = 15% for passenger cars of category M1 and *φ* = 28% for light commercial vehicles of category N1);
*m_l_*—gross vehicle weight rating, kg;
*m_ro_*—vehicle mass in running order, kg;
*m_o_*—additional equipment mass, kg.

The road load power *P_rl_Dyno_i_* simulated on the chassis dynamometer is described by Equation (5):

(5) $$P_{rl}{Dyno}_{i}=\left[f_{0}+f_{1}\cdot {VDyno}_{i}+f_{2}\cdot {\left({VDyno}_{i}\right)}^{2}+\left(1.03\cdot m_{t}\right)\cdot {aDyno}_{i}\right]\cdot \frac{V_{Dynoi}}{3600}$$

where

*P_rl_Dyno_i_*—dyno road load power at time *t_i_*, kW;
*VDyno_i_*—dyno speed at time *t_i_*, km/h;
*m_t_*—test vehicle mass, kg;
*aDyno_i_*—dyno acceleration at time *t_i_*, m/s^2^;
*f*_0_, *f*_1_, *f*_2_—road load coefficients of the tested vehicle, respectively in N, N/(km/h), and N/(km/h)^2^.

**Figure 2:**
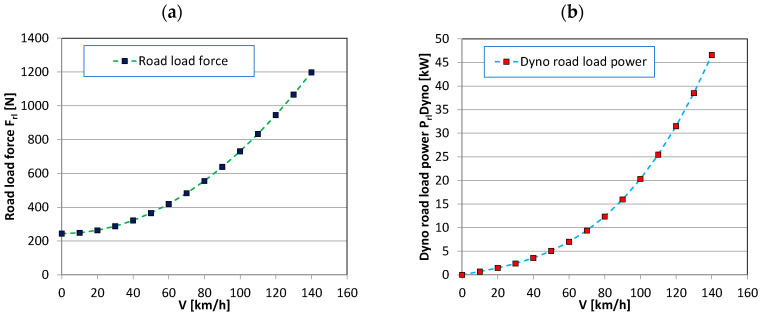
Comparison of the curves of (**a**) road load force *F_rl_* and (**b**) dyno road load power *P_rl_Dyno* as a function of constant vehicle speeds applied in chassis dynamometer testing.

**Figure 3:**
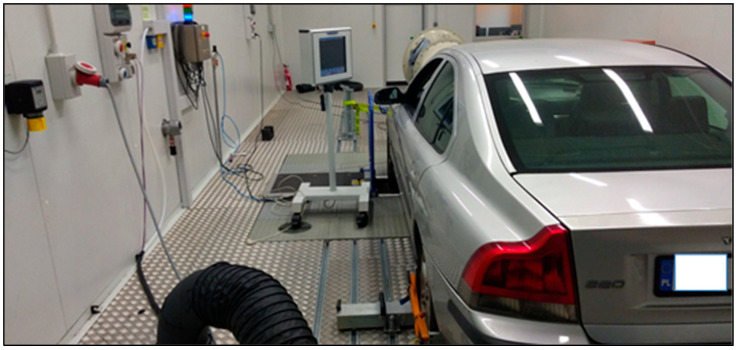
Tested vehicle on the test bench.

The relationship between the road load force and road load power simulated on the chassis dynamometer as a function of constant vehicle speeds is illustrated in Figure 2. Figure 3 presents a photograph of the tested vehicle on the test bench.

**Figure 4:**
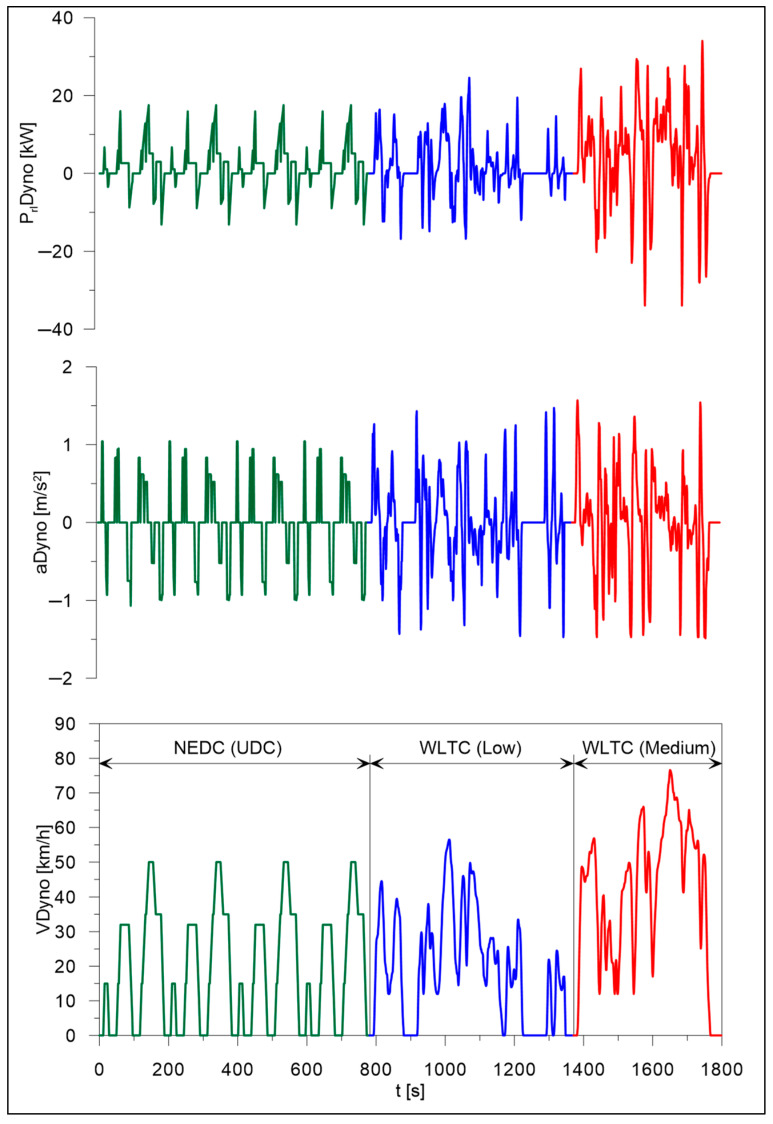
Speed, acceleration, and road load power profiles for the test cycles conducted on the chassis dynamometer 2WD AVL ROADSIM 48″ (AVL List GmbH, Graz, Austria). The green, blue, and red lines indicate the NEDC (UDC), WLTC Low, and WLTC Medium phases, respectively.

The tests were conducted using urban phases of the NEDC and WLTC driving cycles, namely UDC, Low, and Medium Detailed characteristics of the driving cycles used are presented in Table 3. The speed profiles of the test cycles are illustrated in Figure 4. Additionally, the figure presents variations in acceleration a and road load power *P_rl_Dyno* as a function of time. As shown in Figure 4, the highest values of road load power occur mainly during acceleration phases and at higher vehicle speeds, especially in the WLTC Medium phase. In contrast, stop periods and low-speed operation are characterized by substantially lower or near-zero road load power. These time-resolved profiles were used to capture the transient load conditions applied to the CNG powertrain during chassis dynamometer testing.

Exhaust emission measurements were carried out using a constant volume sampling (CVS) system (AVL CVS i60). Prior to each test, a calibration procedure of the exhaust gas analysis system was performed. During the test cycles, exhaust gases diluted with air were directed to gas analyzers, where modal analysis of diluted exhaust gas concentrations was performed using the AVL AMA i60 exhaust gas analysis system. After completion of each test, the emission rates of the following exhaust gas components—HC, CH_4_, NO_X_, CO, and CO_2_—were calculated based on the measured concentrations in diluted exhaust gases and the diluted exhaust gas flow rate, in accordance with Equation (6):

(6) $$m_{gas,i}=\frac{\rho_{gas}\cdot c_{gas,i}\cdot q_{mew,i}}{1,000,000}$$

where

*m_gas,i_*—mass emission rate of the exhaust gas component (CO, HC, NO_X_, CO_2_) at the i-th measurement point, g/s;
*ρ_gas_*—density of the compound in g/L under standard conditions (273.15 K (0 °C) and 101.325 kPa);
*c_gas,i_*—measured concentration of the gaseous component in the exhaust gases at the i-th measurement point, ppm;
*q_mew,i_*—measured exhaust gas flow rate at the *i*-th measurement point, L/s.

The concentration of the gaseous compound in the diluted exhaust gases was corrected taking into account its concentration in the dilution air, in accordance with Equation (7):

(7) $$C_{i}=C_{e}-C_{d}\cdot \left(1-\frac{1}{DF}\right)$$

where

*C_i_*—volume concentration of gaseous component *i* in the diluted exhaust gases, corrected for its concentration in the dilution air, ppm;
*C_e_*—measured volume concentration of gaseous component *i* in the diluted exhaust gases, ppm;
*C_d_*—volume concentration of gaseous component *i* in the dilution air, ppm;
*DF*—dilution factor.

The dilution factor was calculated using Equation (8):

(8) $$DF=\frac{9.5}{C_{{CO}_{2}}+\left(C_{HC}+C_{CO}\right)\cdot 10^{-4}}$$

where

*C_CO_*__2__—CO_2_ concentration in the diluted exhaust gases, %;
*C_HC_*—HC concentration in the diluted exhaust gases, ppm;
*C_CO_*—CO concentration in the diluted exhaust gases, ppm.

The mass *m_gas_* of each gaseous compound emitted by the vehicle during the test was calculated using the following reference densities (at 273.15 K (0 °C) and 101.325 kPa) [37]:

- For CO; *ρ_CO_* = 1.25 g/L;
- For CO_2_; *ρ_CO_*__2__ = 1.964 g/L;
- For NO_X_; *ρ_NO_X__* = 2.05 g/L,
- For HC and CH_4_; *ρ_HC_* = 0.716 g/L.

The average emission of the exhaust gas component *egas* for a given phase (or the entire test cycle) was calculated using Equation (9):

(9) $$egas=\frac{\sum_{i=1}^{n}m_{gasi}\cdot K_{h}}{d}$$

where

*egas*—average emission of the exhaust gas component, g/km;
$K_{h}$—humidity correction factor applied in the calculation of NO_X_ emissions;
d—cycle distance, km.

To ensure proper temporal alignment between vehicle operating parameters and exhaust emissions, all datasets were synchronized using the initial alignment of the CO_2_ concentration signal with vehicle speed, acceleration, and road load power. As all remaining exhaust gas components were measured simultaneously with CO_2_, their synchronization with the vehicle kinematic parameters was performed based on the aligned CO_2_ signal. Laboratory measurements were acquired at a sampling frequency of 10 Hz, while on-road measurements were recorded at 1 Hz. The same synchronization procedure was applied to both laboratory and on-road datasets, thereby ensuring consistent assignment of instantaneous emission values to the corresponding vehicle operating conditions. During on-road validation, speed, acceleration, and *P_rl_* served exclusively as predictor variables for evaluating the models trained on laboratory data.

To estimate the uncertainty of pollutant emission measurements, the error propagation method was applied. This approach assumes random and uncorrelated errors and has been described, among others, in [39]. The uncertainty of emission measurements at the test facility did not exceed ±5% [21]. The tests were conducted in a climatic chamber at an ambient temperature of 21 °C ± 1 °C and a relative humidity of 60% ± 2%. The parameters of the natural gas used to fuel the engine of the tested vehicle are presented in Table 4.

### 2.2. On-Road Testing

**Figure 5:**
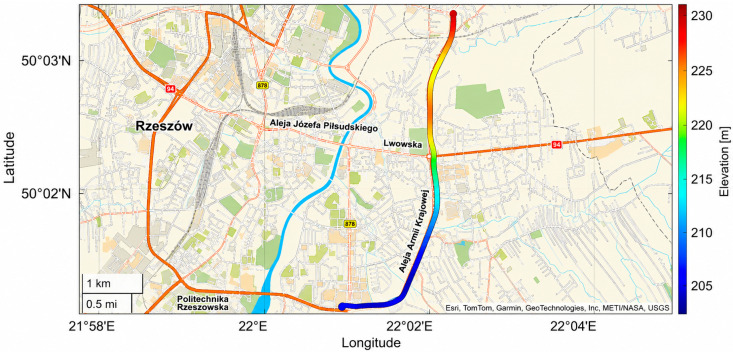
Route map of the RDE tests with the elevation profile indicated.

On-road tests were conducted over a route of approximately 10 km within the urban area of Rzeszow (Figure 5). The tests were performed twice under hot-start conditions (the engine coolant temperature at the beginning of each test was 85 ± 5 °C), under similar ambient temperature conditions of approximately 20 ± 2 °C. The characteristics of the on-road test cycle are presented in Table 5. Emission measurements were carried out using PEMS, specifically the HORIBA OBS-2200 (HORIBA, Ltd., Kyoto, Japan), the specifications of which are summarized in Table 6. Prior to each test, calibration of the exhaust gas analyzers was performed for the following pollutants: CO_2_, CO, HC, and NO_X_. Vehicle speed data were recorded using a GPS system integrated into the PEMS unit. The exhaust gas analysis system did not include CH_4_ analyzer, which constitutes a limitation in the case of natural gas-fueled vehicles. Therefore, CH_4_ emissions under on-road conditions were estimated using the regression models described in Section 2.3. The results and their discussion are presented in Section 3.

The first test run was conducted outside peak traffic hours, while the second was performed during peak hours. The analysis of driving cycle parameters indicates clear differences between the two runs, both in terms of driving dynamics and traffic conditions. For the first run, the value of va_pos95_ was 11.68 m^2^/s^3^ and RPA = 0.211 m/s^2^. These values indicate moderate driving dynamics, with a relatively low number of intensive acceleration events. This is also confirmed by the relatively high average speed of 34.9 km/h and the shorter travel time (1088 s). Such a profile suggests smoother traffic conditions, with fewer stops and more gradual speed variations, which is characteristic of off-peak driving. In contrast, the second run is characterized by higher values of dynamic indicators: va_pos95_ = 12.75 m^2^/s^3^ and RPA = 0.246 m/s^2^. This reflects more frequent and more intense acceleration events, indicating a more dynamic, traffic-induced driving pattern. At the same time, the average speed is significantly lower (24.2 km/h), and the travel time is considerably longer (1561 s). A comparison of the two runs shows that the first represents a more stable and smooth driving pattern, whereas the second reflects conditions associated with higher traffic intensity. The higher values of va_pos95_ and RPA in the second run, despite the lower average speed, confirm the increased intensity of acceleration processes.

**Figure 6:**
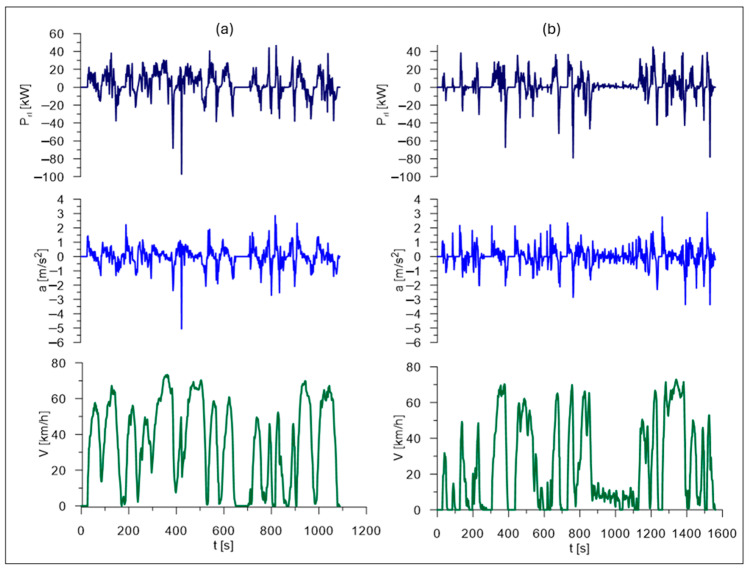
Time histories of speed, acceleration, and road load power: (**a**) first on-road test, (**b**) second on-road test.

The speed profiles of the on-road test cycles are presented in Figure 6. The plots also show variations in acceleration a and road load power *P_rl_* determined for the analyzed conditions. The time histories presented in Figure 6 show that the second on-road test included more frequent stop-and-go operation and more pronounced acceleration events than the first test. These sections correspond to periods of increased road load power and higher transient power demand. In contrast, the flat parts of the speed profile indicate vehicle stops, mainly related to urban traffic conditions such as intersections and traffic lights.

Considering that the road load power in chassis dynamometer testing accounts for drivetrain losses, the road load power acting on the vehicle during on-road tests was also determined with these losses taken into account. These losses were represented by the average drivetrain efficiency coefficient η, and the road load power was calculated using Equation (10):

(10) $$P_{rl}=\frac{V\cdot a\cdot m_{r}\cdot \left(1+\delta \right)+m_{r}\cdot g\cdot w\cdot V+m_{r}\cdot g\cdot f_{t}\cdot V+0.6\cdot A_{d}\cdot c_{d}\cdot V^{3}}{\eta }$$

where

*V*—vehicle speed, m/s;
*a*—vehicle acceleration, m/s^2^;
*δ*—rotational mass coefficient, (δ = 1.03);
*m_r_*—test vehicle mass in on-road tests, kg (*m_r_* = 1850 kg);
*A_d_*—frontal area of the vehicle, m^2^ (A_d_ = 2.58 m^2^);
*c_d_*—aerodynamic drag coefficient in the longitudinal direction, (c_d_ = 0.28);
*f_t_*—rolling resistance coefficient, (f_t_ = 0.012);
*g*—gravitational acceleration, m/s^2^ (g = 9.81 m/s^2^);
w—road grade, %;
*η*—drivetrain efficiency, % (η = 90%).

### 2.3. Development of Emission Models

**Figure 7:**
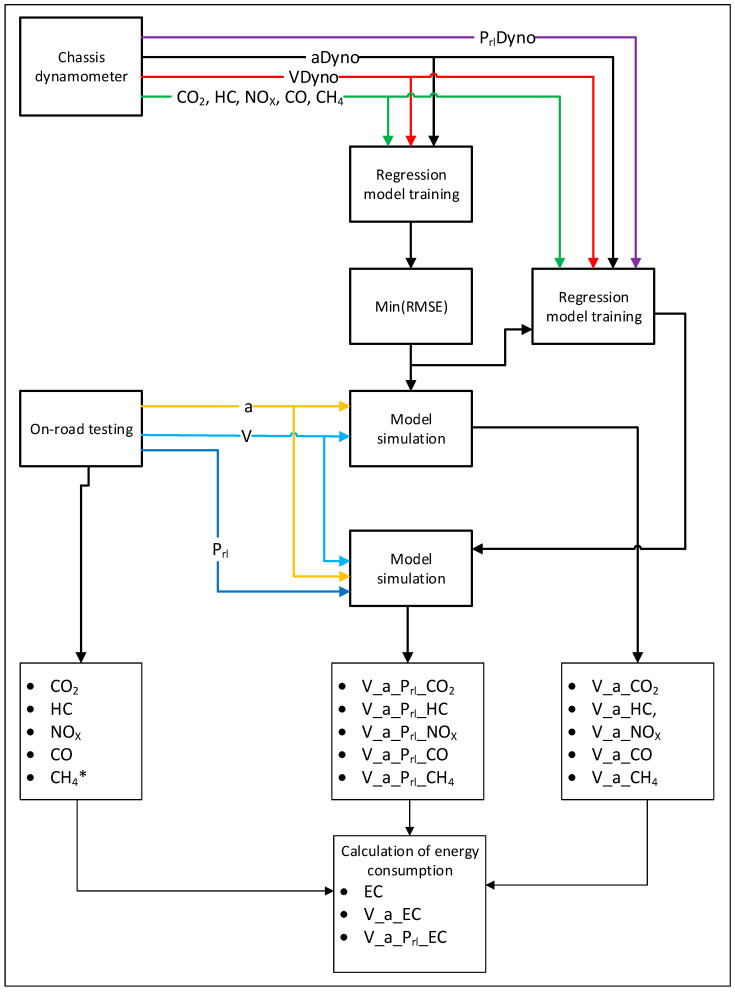
Schematic of the adopted research methodology. * Methane emissions estimated based on the percentage share of CH_4_ in total hydrocarbon (HC) emissions determined from laboratory tests.

The development and validation of exhaust emission models involved several stages, ranging from data preparation and model training to validation under real-world conditions, as illustrated in the schematic of the adopted research methodology shown in Figure 7.

**Figure 8:**
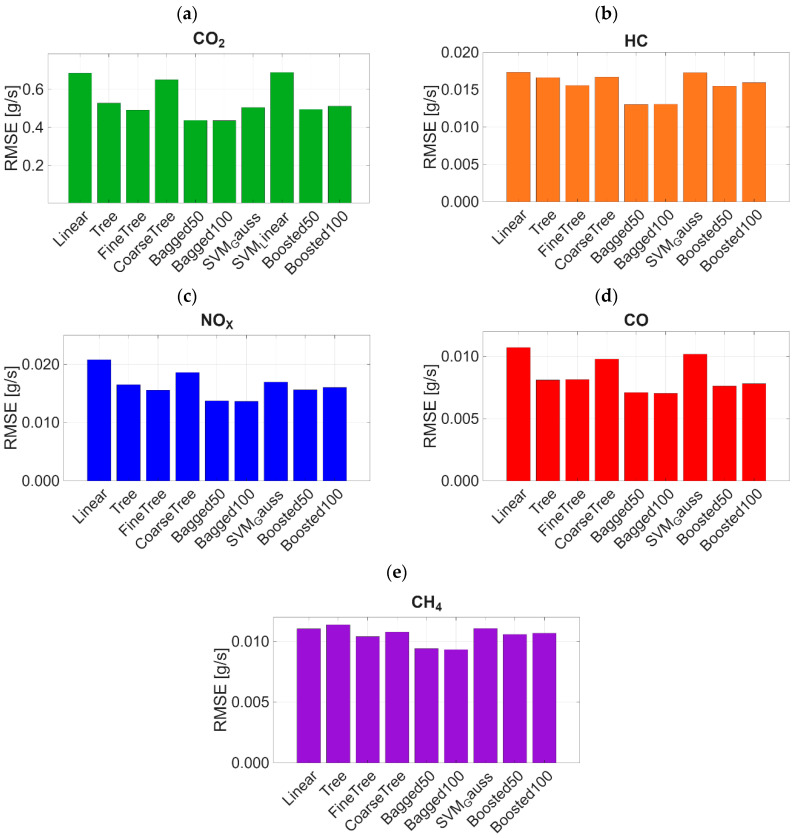
Comparison of RMSE values for the analyzed models: (**a**) CO_2_, (**b**) HC, (**c**) NO_X_, (**d**) CO, and (**e**) CH_4_.

Based on the results obtained from chassis dynamometer testing, and considering approaches applied by other authors, two input variables were selected: vehicle speed (*VDyno*) and vehicle acceleration (*aDyno*). These variables were used as predictors in the training process of the regression models. The dependent variables were the emissions of selected exhaust gas components, namely CO_2_, HC, NO_X_, CO, and CH_4_. During model development, cross-validation was applied, in which 80% of the data, randomly selected, were assigned to the training set, while the remaining 20% were used for validation. Model performance was evaluated using the root mean square error (RMSE) metric. The RMSE values for the individual regression models obtained in the Regression Learner environment (MATLAB) are presented in Figure 8 for the analyzed exhaust gas components.

The analysis of the results presented in Figure 8 showed that, for all considered exhaust gas components, the lowest RMSE values were achieved by Bagged Trees models, which were therefore selected for further analyses. Hyperparameter settings and structural characteristics of the bagged-tree regression models developed for exhaust emission prediction are summarized in Table 7. The developed bagged-tree regression models demonstrated consistent hyperparameter settings across all predicted emission components, ensuring methodological comparability between the analyzed variants. Each model was constructed as an ensemble of 30 regression trees using bootstrap aggregation with a minimum leaf size of eight observations, while the split criterion was based on minimization of mean squared error (MSE). No input feature normalization was applied during model training.

The structural complexity of the resulting trees varied depending on the modeled emission component. Among all considered outputs, the CO_2_ models exhibited the highest complexity, reaching the greatest tree depths and largest number of split nodes, which indicates stronger nonlinear dependencies between the predictor variables and carbon dioxide emissions. Intermediate structural complexity was observed for CH_4_ and CO prediction models, while HC and NO_X_ models required comparatively simpler tree structures.

A comparison of the *V_a* and *V_a_P_rl_* variants revealed only minor differences in tree depth and split count, suggesting that both predictor configurations produced models of comparable structural complexity. This indicates that performance differences between the two variants are likely associated with the predictive information carried by the input variables rather than substantial differences in model architecture.

**Figure 9:**
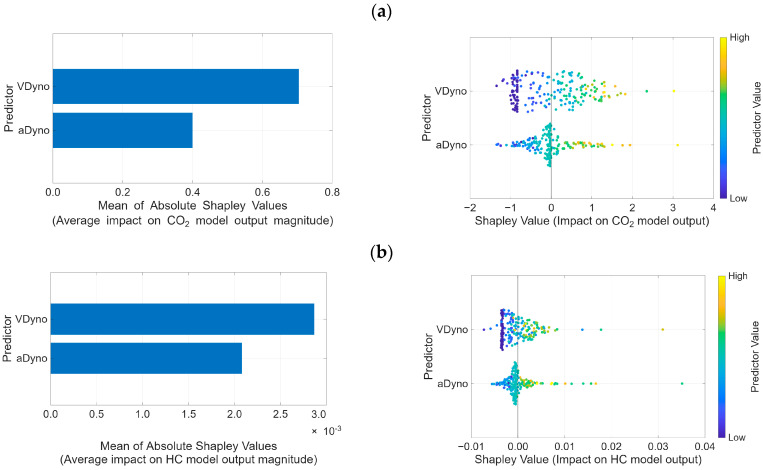

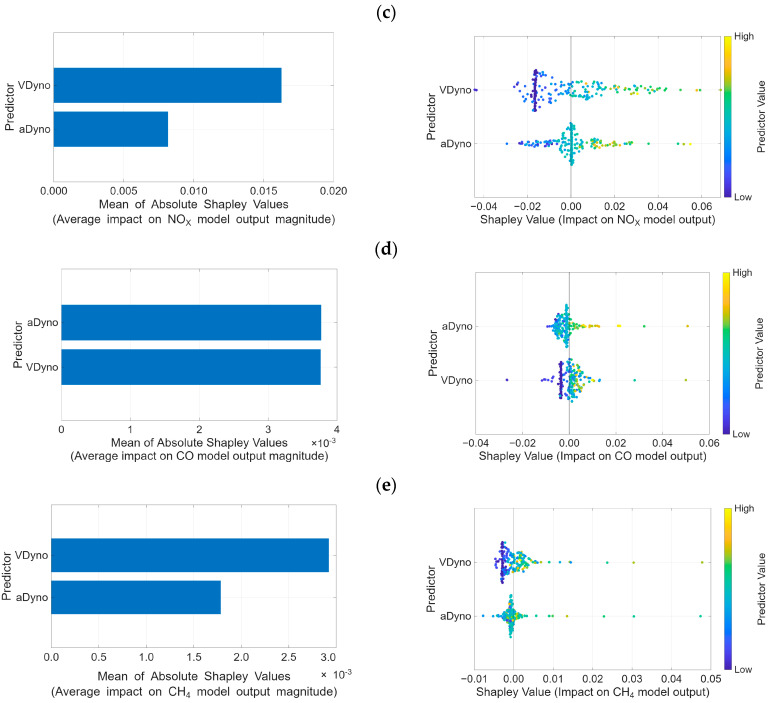
Analysis of the influence of input variables on the prediction results of exhaust emission models using SHAP values: (**a**) CO_2_, (**b**) HC, (**c**) NO_X_, (**d**) CO, and (**e**) CH_4_; the left panels present mean absolute SHAP values (predictor importance), while the right panels show scatter plots illustrating the direction and magnitude of the influence of the variables (*VDyno*, *aDyno*) on the predicted values.

Linear models were characterized by higher error values, whereas models based on single trees and boosting methods achieved intermediate results. No significant improvement in model performance was observed with an increasing number of trees, indicating that the models had reached a saturation level. To interpret the influence of the input variables on prediction results, an analysis based on SHAP values was performed. The results are presented as bar charts (mean absolute SHAP values) and scatter plots (Figure 9). The analysis for models using two predictors (*VDyno* and *aDyno*) showed that vehicle speed was the dominant variable influencing the prediction of emissions for most exhaust gas components. For CO_2_, HC, NO_X_, and CH_4_, the SHAP values for *VDyno* were significantly higher than those for *aDyno*, indicating the greater importance of vehicle speed in the modeling process. An exception was CO emissions, for which the influence of both variables was comparable. The SHAP scatter plots confirmed this relationship, showing an increasing positive contribution with increasing vehicle speed, whereas the effect of acceleration was more dispersed and less clearly defined.

**Figure 10:**
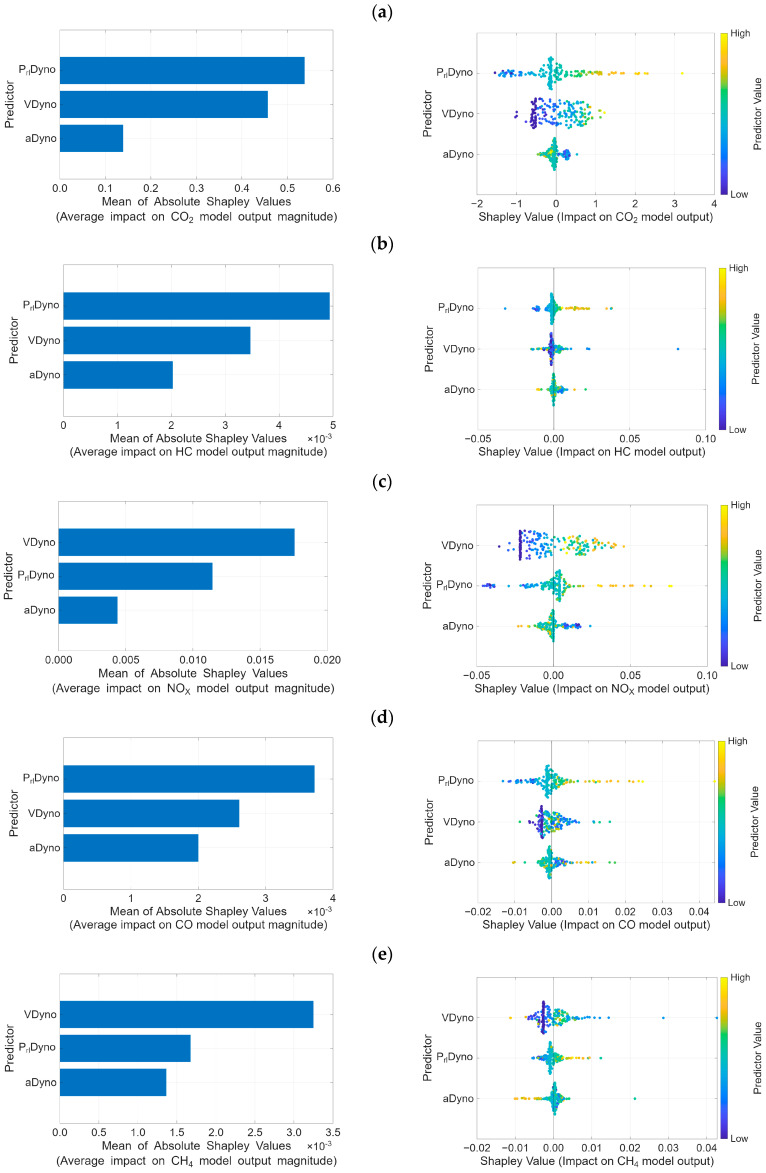
Analysis of the influence of input variables on the prediction results of exhaust emission models using SHAP values for models extended with an additional predictor, road load power (*P_rl_Dyno*): (**a**) CO_2_, (**b**) HC, (**c**) NO_X_, (**d**) CO, and (**e**) CH_4_; the left panels present mean absolute SHAP values (predictor importance: *VDyno*, *aDyno*, *P_rl_Dyno*), while the right panels show scatter plots illustrating the direction and magnitude of the influence of individual variables on the predicted values.

In the next stage, the set of predictors was extended by including an additional variable—road load power (*P_rl_Dyno*). The SHAP analysis for the three-parameter models (Figure 10) showed that *P_rl_Dyno* became one of the key predictors, particularly for CO_2_, HC, and CO, where its influence was comparable to or greater than that of vehicle speed. At the same time, the importance of acceleration (*aDyno*) decreased markedly in all analyzed cases. For NO_X_, vehicle speed remained the dominant predictor, whereas for CH_4_ a more balanced contribution of *VDyno* and *P_rl_Dyno* was observed. The scatter plots also indicated more structured relationships between predictor values and emissions in the extended models, suggesting a better representation of the underlying physical phenomena.

The observed reduction in the SHAP importance of acceleration after the inclusion of *P_rl_* can be attributed to the fact that road load power inherently combines inertial effects with external resistive forces, thereby providing a more complete representation of instantaneous powertrain load than acceleration alone.

The trained models were subsequently tested using data obtained from real-world driving in the on-road cycle (Figure 6). The input data consisted of vehicle speed (*V*), acceleration (*a*), and road load power (*P_rl_*) profiles. The simulation results for models using the variables (*V*, *a*) and (*V*, *a*, *P_rl_*) were compared with the actual emission values recorded during the on-road tests and are presented in Section 3.2.

The average energy consumption values were calculated based on emission factors, determined using the carbon balance method, in accordance with the following equation:

(11) $$EC=\frac{0.1336}{\rho_{NG}}\cdot \left(0.749\cdot eHC+0.429\cdot eCO+0.273\cdot {eCO}_{2}\right)\cdot LHV$$

where

*EC*—energy consumption, kWh/100 km;
*ρ_NG_*—natural gas density at normal conditions, kg/m^3^;
*eHC*—average emission of total hydrocarbons, g/km;
*eCO*—average emission of carbon monoxide, g/km;
*eCO*_2_—average emission of carbon dioxide, g/km;
*LHV*—lower heating value of natural gas, kWh/m^3^.

## 3. Results and Discussion

### 3.1. Experimental Results

**Figure 11:**
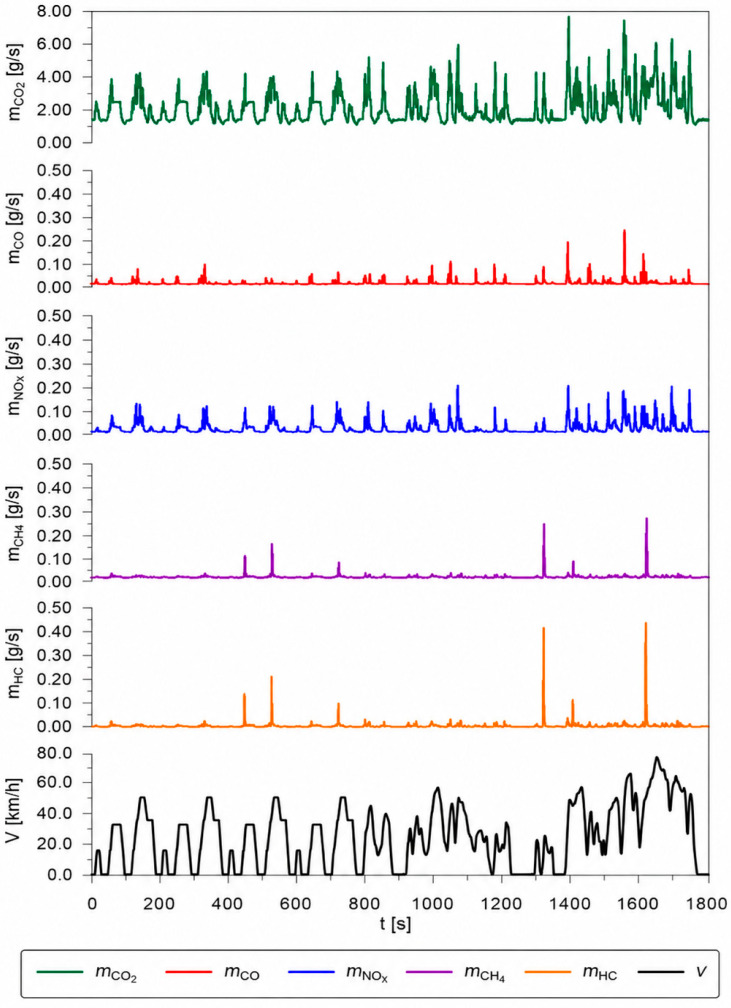
Results of gaseous emission measurements (CO_2_, CO, NO_X_, CH_4_, and HC) during the test cycle of the CNG-fueled vehicle on the chassis dynamometer.

The time-resolved emission records shown in Figure 11 indicate that the highest CO_2_ and NO_X_ emission rates generally occur during acceleration phases and periods of increased road load power. This confirms the strong relationship between transient engine load and the formation of these components. In contrast, HC, CH_4_, and CO emissions show more irregular behavior, which may result from transient combustion conditions and the operation of the aftertreatment system.

**Figure 12:**
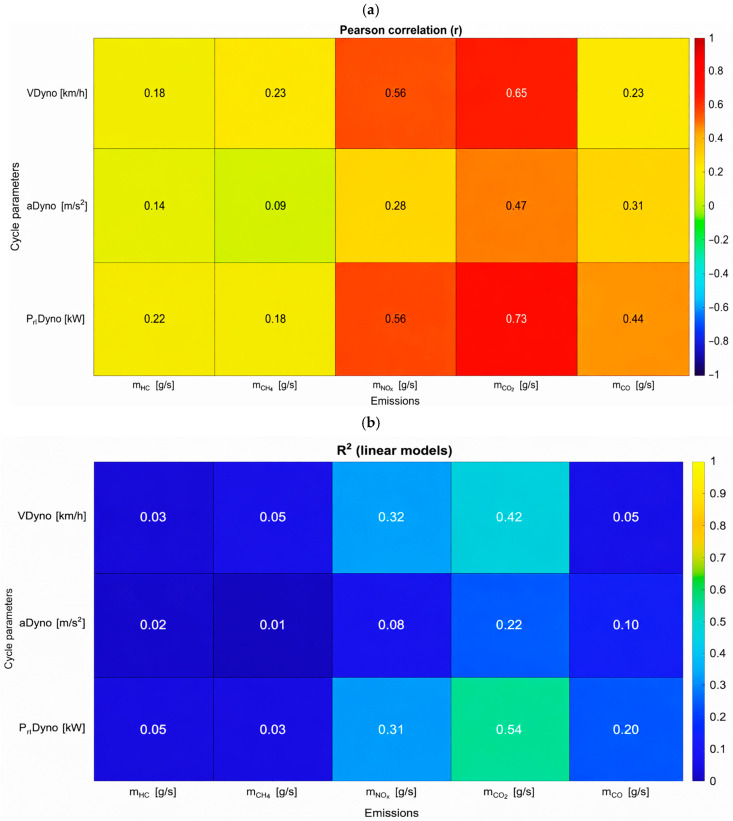
Pearson correlation coefficients (**a**) and R^2^ values (**b**) between vehicle speed *VDyno*, acceleration *aDyno* and dyno road load power *P_rl_Dyno* and the emission rates of CO_2_, CO, NO_X_, HC, and CH_4_ in laboratory tests.

The values of Pearson correlation coefficients and R^2^ between vehicle speed *VDyno*, acceleration *aDyno*, road load power *P_rl_Dyno*, and the measured emission rates of the analyzed pollutants (HC, CH_4_, NO_X_, CO_2_, and CO) are presented in Figure 12. It can be observed that the highest correlation coefficient (r = 0.73; R^2^ = 0.54) occurs between road load power *P_rl_Dyno* and the emission rate of CO_2_. A similarly high value of R^2^ is also found between vehicle speed and CO_2_ emission rate (r = 0.64). Thus, vehicle speed shows the strongest relationship with CO_2_ emissions, as well as with NO_X_ emissions (r = 0.56). The correlation between vehicle speed and the emission rates of CO and CH_4_ is lower but comparable, with R^2^ values of 0.03 and 0.05, respectively. Road load power also exhibits relatively high correlation with NO_X_ emissions (r = 0.56) and CO emissions (r = 0.44). The lowest R^2^ values, indicating weak correlations between *P_rl_Dyno* and HC and CH_4_ emission rates, are 0.05 and 0.03, respectively. For acceleration, the correlation coefficients with emission rates are as follows: 0.14 for HC, 0.09 for CH_4_, 0.28 for NO_X_, 0.47 for CO_2_, and 0.31 for CO. These results confirm that, in addition to speed and acceleration, road load power is also an important predictor in emission models.

**Figure 13:**
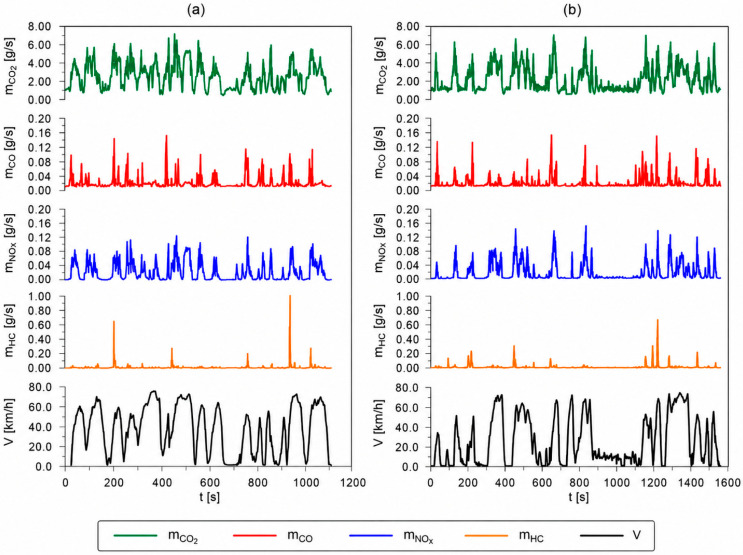
Emission rates of CO_2_, CO, NO_X_, and HC against the speed profile in on-road tests: (**a**) run 1, (**b**) run 2.

The results of the on-road tests are presented in Figure 13, which shows the emission rates of the analyzed pollutants for run 1 (Figure 13a) and run 2 (Figure 13b). The on-road emission profiles shown in Figure 13 further confirm the influence of real traffic conditions on emission behavior. Sections with rapid acceleration and increased road load are accompanied by visible increases in CO_2_ and NO_X_ emission rates, whereas stop periods and low-speed operation reflect typical urban stop-and-go driving. The second test contains more such transient sections, which is consistent with its higher RPA and va_pos95_ values (Table 5).

It should also be noted that the tested Euro 3 CNG vehicle was not equipped with a start–stop system. Consequently, emission events recorded during vehicle stops and urban stop-and-go operation correspond to continuous engine idling conditions. The potential influence of start–stop functionality on real-world emissions may constitute an interesting direction for future studies.

### 3.2. Results of Modeling Studies

**Figure 14:**
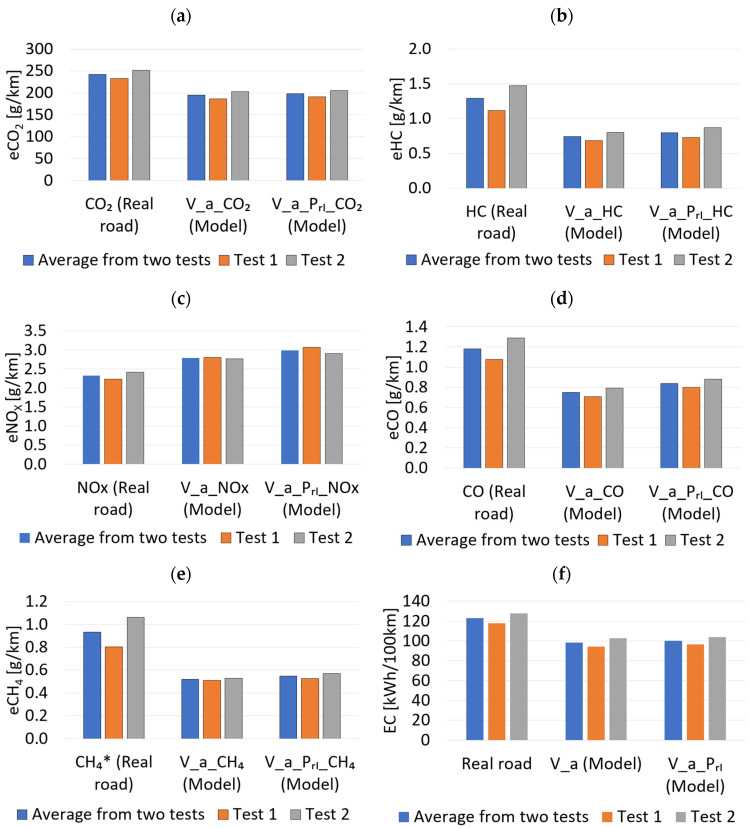
Comparison of average exhaust emission values and energy consumption obtained during the on-road tests and values predicted by the regression models: (**a**) CO_2_, (**b**) HC, (**c**) NO_X_, (**d**) CO, (**e**) CH_4_ (CH_4_*—estimated methane emissions), and (**f**) EC.

**Figure 15:**
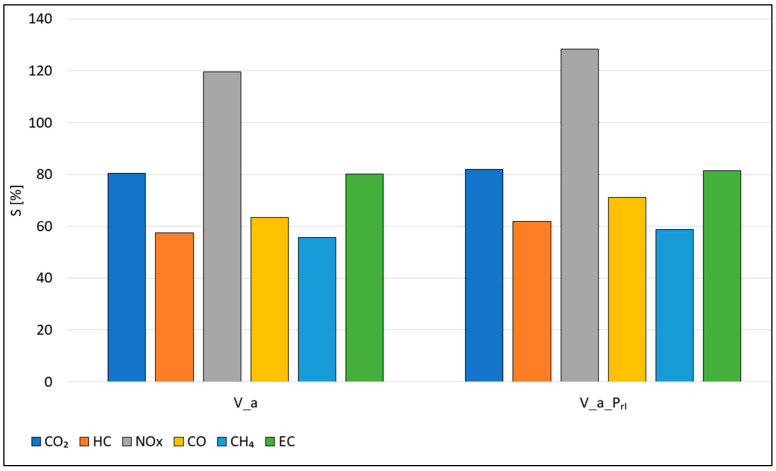
Values of the prediction accuracy index S for models using two predictors (V_a) and three predictors (*V_a_P_rl_*).

Emission models developed from laboratory data were applied to predict emissions using predictors derived from on-road testing. The evaluation of the impact of the additional predictor (*P_rl_*) on the emission modeling results for the analyzed pollutants was based on a comparison of the average emission factors measured in on-road tests with the values predicted by the models (Figure 14), the prediction agreement index S (Figure 15), the correlation coefficient r, the coefficient of determination R^2^, the mean absolute error (MAE), the root mean square error (RMSE), and the mean absolute percentage error (MAPE) (Table 8).

Figure 14 presents a comparison of the average emissions of exhaust gas components and energy consumption obtained during the on-road tests with the values predicted by the regression models. The experimental data are denoted as *Real road*. The models labeled *V_a* use two predictors (vehicle speed and acceleration), whereas *V_a_P_rl_* models additionally include *P_rl_* as a third predictor.

For CO_2_ emissions (Figure 14a), both models reproduce the general emission trend but consistently underestimate the measured values. The average measured CO_2_ emission is approximately 240 g/km, whereas the *V_a_CO*_2_ model predicts about 195 g/km (≈−19%) and the *V_a_P_rl_*_*CO*_2_ model about 200 g/km (≈−17%). The inclusion of *P_rl_* slightly improves the agreement with the experimental data, reducing the underestimation by approximately 2 percentage points. These discrepancies are consistent with literature reports indicating higher CO_2_ emissions under real-world driving conditions compared to laboratory-based procedures such as WLTP [40,41].

For HC emissions (Figure 14b), the models show good qualitative agreement with the experimental data but also tend to underestimate emissions. The average real-world HC emission is about 1.3 g/km, compared to approximately 0.75 g/km for *V_a_HC* (≈−42%) and 0.8 g/km for *V_a_P_rl_*_*HC* (≈−38%). The inclusion of *P_rl_* provides a modest improvement, although the underestimation remains substantial.

In the case of NO_X_ emissions (Figure 14c), both models overestimate the measured values. The average experimental emission is approximately 2.3 g/km, whereas *V_a_NO_X_* and *V_a_P_rl__NO_X_* predict approximately 2.7–2.8 g/km, corresponding to an overestimation of about +17–22%. Unlike the other analyzed pollutants, the inclusion of *P_rl_* did not improve the NO_X_ prediction accuracy and resulted in a higher S index value, indicating increased model overestimation. This suggests that NO_X_ formation is influenced by additional thermodynamic and combustion-related factors that were not included in the present simplified model structure. In particular, parameters such as catalyst temperature, air–fuel ratio, engine speed, instantaneous engine load, and exhaust gas temperature may have a stronger influence on NO_X_ formation than vehicle road load alone. Therefore, accurate NO_X_ prediction may require a more complex modeling approach based on an extended set of input variables.

For CO emissions (Figure 14d), both models reproduce the emission level with relatively good accuracy. The measured average value is about 1.2 g/km, while *V_a_CO* predicts approximately 0.75 g/km (≈−38%) and *V_a_P_rl__CO* about 0.85 g/km (≈−29%). Although both models underestimate emissions, the inclusion of *P_rl_* improves agreement with the experimental data.

For CH_4_* emissions (Figure 14e), CH_4_ during RDE operation was not measured directly due to the lack of a dedicated methane analyzer in the HORIBA OBS-2200 system. Instead, CH_4_* was estimated based on the methane fraction in total hydrocarbon emissions determined from chassis dynamometer tests. The CH_4_/HC emission share coefficient for the tested vehicle, derived from five independent tests (*n* = 5), was 0.7265 ± 0.0340 (mean ± standard deviation), with a median of 0.7392 and a range of 0.6626–0.7591. The mean value was applied to estimate CH_4_ emissions in road tests for the tested vehicle based on measured HC concentrations, assuming a constant CH_4_/HC relationship. The results indicate low variability of the coefficient and good repeatability across the performed measurements. A similar share of CH_4_ emissions relative to total HC emissions was reported by Bielaczyc et al. [42] in tests of a natural gas-powered passenger vehicle conducted under the NEDC driving cycle. Both models therefore reflect the ability to reproduce laboratory-derived HC–CH_4_ relationships under real-world operating conditions.

The obtained results indicate a systematic underestimation of CH_4_*, with an average experimental value of approximately 0.95 g/km compared to 0.50 g/km for the *V_a_CH*_4_ model (≈−47%) and 0.55 g/km for the *V_a_P_rl_*_*CH*_4_ model (≈−42%). The inclusion of the additional predictor leads only to a minor improvement, suggesting limited capability of the adopted input variables to fully capture methane formation dynamics in CNG engine operation.

Overall, the inclusion of *P_rl_* generally improves model performance for most analyzed components, although NO_X_ emissions constitute a notable exception.

The predicted values—particularly for CO, HC, NO_X_, and CH_4_—are higher than those typically estimated using commonly applied emission inventory tools such as COPERT [43]. It should be emphasized that COPERT represents a macroscopic, fleet-average emission inventory methodology based on aggregated emission factors, whereas the present study develops vehicle-specific, instantaneous emission models derived from transient measurement data and machine learning techniques. Therefore, a direct quantitative comparison between both approaches is not strictly valid, and the COPERT values are used here only as a general reference range for urban CNG passenger cars, rather than as a validation benchmark.

For a representative CNG passenger car (large segment) operating under urban conditions at an average speed of approximately 25 km/h, COPERT predicts NO_X_ emissions on the order of ≈0.18 g/km, while CO, HC, and CH_4_ are typically within ~20–80 g/100 km (CO), ~5–30 g/100 km (HC), and ~10–40 g/100 km (CH_4_), depending on driving conditions and model assumptions. Similarly, higher emission rates have been reported in other studies conducted for vehicles powered by natural gas [26,44,45].

Although the regression models developed in this study do not fully eliminate the deviation from measured values, they consistently provide estimates much closer to the experimental data than COPERT-based calculations, especially for CH_4_, but also for CO, HC, and NO_X_.

The prediction accuracy of the developed models was evaluated using the *S* index, defined as the percentage ratio of the emission value (or energy consumption) determined by the model to the emission value obtained during the on-road RDE tests (Equation (12)).

(12) $$S_{i}=\frac{e_{i}Model}{e_{i}RDE}\cdot 100\%$$

where

*S_i_*—prediction accuracy index for component i, %;
*e_i_Model*—average emission of component i determined by the model for both runs, g/km;
*e_i_RDE*—average emission of component i determined in the RDE tests for both runs, g/km.

A value of the index equal to 100% indicates perfect reproduction of real emissions, whereas values below or above this threshold indicate underestimation or overestimation of the results by the model, respectively. Figure 15 presents the values of the *S* index for models using two predictors (*V*, *a*) and three predictors (*V*, *a*, *P_rl_*). The analysis of the results indicates that, for all analyzed exhaust gas components, the models show a tendency to underestimate emissions, as confirmed by *S* values lower than 100%.

For the two-predictor models (V_a), the values of the *S* index are approximately 80% for CO_2_, 57% for HC, 120% for NO_X_, 64% for CO, 55% for CH_4_*, and 80% for EC.

This indicates that the models reproduce CO_2_ emissions relatively well, whereas significant underestimation is observed for HC, CO, and CH_4_*. An exception is NO_X_ emissions, for which the model shows a tendency toward overestimation.

Extending the models with an additional predictor in the form of road load power (*V_a_P_rl_*) leads to improved agreement for most of the analyzed components. In particular, an increase in the S index is observed for HC (to approximately 61%), CO (to approximately 71%), and CH_4_* (to approximately 59%), indicating a reduction in underestimation error. An improvement was also noted for CO_2_ and EC (to approximately 82%), whereas for NO_X_ a further increase in overestimation was observed (to approximately 128%).

The obtained results indicate that including road load power as an additional predictor improves the ability of the models to reproduce emissions of most exhaust gas components, particularly those strongly dependent on engine load conditions. This effect can be explained by the fact that *P_rl_* more explicitly represents instantaneous traction power demand resulting from aerodynamic drag, rolling resistance, and road grade, which are only indirectly reflected in speed- and acceleration-based descriptors. Consequently, *P_rl_* provides a more physically grounded approximation of engine operating conditions, improving the model’s sensitivity to transient load variations that govern combustion quality.

At the same time, the deterioration of results for NO_X_ suggests that the mechanisms governing the formation of this component are not fully captured by the adopted set of input variables. NO_X_ formation is strongly influenced by high-temperature combustion conditions and aftertreatment behavior, which may exhibit a different dependency structure than load-driven incomplete combustion phenomena. Therefore, the applied predictors appear more effective in describing load-sensitive emissions such as CO, HC, CH_4_*, CO_2_, and EC, while being less representative for NO_X_ formation pathways.

In summary, the S index confirms that the developed models exhibit varying prediction accuracy depending on the analyzed exhaust gas component. The best agreement was obtained for CO_2_ and EC, whereas the largest deviations were observed for CH_4_* and NO_X_. The inclusion of an additional predictor (*P_rl_*) leads to an overall improvement in prediction quality; however, it does not eliminate all discrepancies between the model results and the experimental data.

It should be emphasized that the differences observed between the on-road test results and the model predictions may stem from several important factors. The model outputs were generated using regression models trained on laboratory (chassis dynamometer) data, whereas the reference values were obtained from real-world, on-road measurements.

The predictors used in the models—vehicle speed (*V*) and acceleration (*a*)—were derived from on-road test data and represent quantities that can be measured or calculated with high accuracy. In contrast, the additional predictor, road load power (*P_rl_*), was estimated under on-road conditions using a simplified approach that does not fully capture the complexity of real-world influences.

In particular, actual vehicle load is affected by factors that cannot be reliably reproduced based solely on speed and acceleration profiles. These include wind gusts, variations in aerodynamic drag, cornering resistance, local changes in the rolling resistance coefficient, road surface micro- and macro-roughness, fluctuations in road grade, and varying vehicle operating conditions (e.g., changes in vehicle mass, operation of auxiliary systems, or engine control strategies). As a result, road load power cannot be determined with complete accuracy using only basic vehicle motion parameters.

Despite these limitations, incorporating road load power as an additional predictor significantly improves the models’ ability to reproduce exhaust emissions. This is because *P_rl_* provides a synthetic representation of powertrain load, effectively aggregating the influence of multiple physical phenomena that are not directly captured by speed and acceleration alone.

Consequently, even with the simplified method used to estimate road load power under real-world conditions, its inclusion enhances overall prediction accuracy. Moreover, the proposed approach allows the models to be applied in situations where only basic vehicle motion parameters (*V*, *a*) are available, thereby increasing their practical utility. Nevertheless, it should be acknowledged that the limited accuracy of *P_rl_* estimation under real-world conditions remains one of the sources of discrepancies between model predictions and on-road measurements.

Analysis of the results presented in Table 8 (r, R^2^, MAE, RMSE, MAPE) clearly indicates that the inclusion of road load power as an additional predictor does not degrade the predictive performance of the models under real-world driving conditions, with all statistical metrics remaining at a comparable level. At the same time, despite the lack of consistent improvements in global error indicators, the *V_a_P_rl_* models demonstrate a more coherent reproduction of average emission levels for most analyzed pollutants, indicating better agreement with real-world vehicle operation. This finding should be interpreted as a meaningful physical advantage: *P_rl_* introduces information on vehicle power demand arising from road resistance effects that cannot be fully captured by vehicle speed and acceleration alone. Consequently, although its impact on conventional statistical metrics is limited, the inclusion of *P_rl_* enhances the physical fidelity of the model and improves its ability to represent real driving conditions, which is a key advantage in the context of RDE-based emission modeling.

The obtained results also indicate that the analyzed CNG-powered vehicle maintained stable operating behavior under increased transient load conditions occurring during more dynamic urban driving, which further confirms the importance of including load-related parameters in real-world emission and energy consumption modeling. It should also be noted that the present study focused on transient urban driving conditions and did not include dedicated tests under prolonged constant-load operation, such as sustained uphill driving. Therefore, possible thermal effects associated with long-duration high engine load were not explicitly evaluated and should be addressed in future studies.

## 4. Conclusions

The conducted study demonstrated that the proposed approach, based on regression models trained using chassis dynamometer data, enables effective reproduction of exhaust emissions and energy consumption under real-world driving conditions. A significant advantage of the developed method is the use of laboratory data, characterized by higher measurement accuracy than on-road measurements, which translates into improved model training quality. In addition, this approach enables the inclusion of CH_4_ emissions, the measurement of which under on-road conditions requires advanced PEMS equipment not available in most standard measurement systems, while being particularly important in emission analyses of natural gas-fueled vehicles.

The main conclusions drawn from the conducted analyses are as follows:

- The developed models enabled accurate reproduction of both emission and energy consumption trends for all analyzed components, with the best agreement obtained for CO, CO_2_, and energy consumption;
- The use of laboratory data allowed for the development of models based on more precise and stable measurements, which represents a significant advantage over approaches relying exclusively on on-road data;
- The inclusion of an additional predictor in the form of road load power (*P_rl_*) significantly improved prediction quality, confirming the importance of accounting for powertrain load conditions;
- Despite the simplified method used to determine *P_rl_* under on-road conditions, its application increased the ability of the models to reproduce real-world emissions and energy consumption;
- The developed models also enabled the estimation of CH_4_ emissions without the need for advanced measurement equipment under on-road conditions.

The obtained results justify further research focused on improving methods for estimating vehicle load conditions and integrating additional variables describing real-world operating conditions, which may further enhance model accuracy. However, NO_X_ emissions constituted an important exception, as the inclusion of road load power did not improve prediction accuracy. This indicates the need to incorporate additional combustion- and aftertreatment-related parameters in future model development aimed at improving NO_X_ emission prediction.

Due to the limited number of tested vehicles and driving cycles, the present study should be considered exploratory in nature, and the developed models should be interpreted within the validity range of the analyzed dataset. Nevertheless, the proposed hybrid ML methodology incorporating road load power is scalable and may be extended to larger and more diverse datasets in future research.

## Figures and Tables

**Table 1:** Specification of chassis dynamometer and exhaust emissions measurement equipment [35,36].

Device Name	Type	Measurement Range	Accuracy
Chassis Dynamometer Speed	2WD AVL ROADSIM 48″ *	0–200 km/h	±0.05%
Tractive force	-	0–10 kN	±0.1%
Distance	-	-	0.001%/m
Exhaust gas sampling system	AVL CVS i60	2–20 m^3^/min	±2%
Absolute pressure sensor	-	700–1100 hPa	±0.25% of the set range
Temperature sensor measurement	-	273–373 °K	±1 °K
Humidity sensor measurement range	-	0–100%	±1%
System of exhaust gas analysis	AVL AMA i60	-	-
CO_2_ analyzer	AVL IRD i60 *	0–20%	±2% of the measurement value
CO analyzer	AVL IRD i60	0–5000 ppm	±2% of the measurement value
HC analyzer	AVL FID i60 *	0–1000 ppm	±2% of the measurement value
CH_4_ analyzer	AVL FID i60	0–1000 ppm	±2% of the measurement value
NO and NO_X_	AVL CLD i60 *	0–1000 ppm	±2% of the measurement value

* AVL List GmbH, Graz, Austria.

**Table 2:** Basic technical specifications of the tested vehicle and roller dynamometer settings.

Parameter	Value	Unit
Length/Width/Height	mm	4576/1804/1428
Wheelbase	mm	2715
Curb weight	kg	1600
Engine type	-	spark-ignition (SI)
Fuel	-	CNG
Fuel system	-	MPI, gaseous phase
Engine displacement	cm^3^	2435
Maximum engine power/speed	kW/rpm	103/4500
Maximum torque/speed	Nm/rpm	220/3750
Drive axle	-	Front
Number of gears (manual transmission)	-	5
Mileage	km	275,000
Tire size	-	205/55 R16
Road load force *F_rl_* coefficients (target)	N; N/(km/h); N/(km/h)^2^	f_0_ = 247.5; f_1_ = 0; f_2_ = 0.04865
Test vehicle mass *m_t_*	kg	1768

**Table 3:** Characteristics of the driving cycles used in laboratory tests [37,38].

Parameter	Unit	NEDC–UDC	WLTC-Low (Class 3b)	WLTC-Medium (Class 3b)
Duration	s	780	589	433
Stop duration	s	228	156	43
Distance	km	3.976	3.095	4.756
Maximum speed	km/h	50	56.5	76.6
Average speed (including stops)	km/h	18.35	18.9	39.5
Average driving speed (excluding stops)	km/h	25.93	25.7	44.5
Maximum acceleration	m/s^2^	1.042	1.47	1.57
Maximum deceleration	m/s^2^	−1.042	−1.47	−1.49
Urban part va_pos95_ *	m^2^/s^3^	7.02	7.95	12.30
Urban part RPA **	m/s^2^	0.131	0.194	0.191

* va_pos95_—95th percentile of positive acceleration and speed; ** RPA—Relative Positive Acceleration.

**Table 4:** Properties of CNG used in driving cycle tests *.

Parameter	Unit	Value
Higher heating value	kWh/m^3^	11.296
Lower heating value	kWh/m^3^	10.191
Lower heating value	MJ/kg	49.050
Density at 15 °C and 101.325 kPa	kg/m^3^	0.748
Stoichiometric air–fuel ratio	kg air/kg CH_4_	17.2
Motor octane number (MON)	-	105
Research octane number (RON)	-	110
CH_4_ concentration	%	96.341
N_2_ concentration	%	0.620
CO_2_ concentration	%	0.231
O_2_ concentration	%	0.000
Ethane concentration	%	1.985
Propane concentration	%	0.596
i-Butane concentration	%	0.094
n-Butane concentration	%	0.088
i-Pentane concentration	%	0.019
n-Pentane concentration	%	0.013
C6+ hydrocarbons concentration	%	0.014

* values specified by the gas supplier.

**Table 5:** Characteristics of the on-road tests.

Parameter	Unit	Test 1	Test 2
Total time	s	1088	1561
Idle time	s	111	320
Total distance	km	10.541	10.478
Average speed (including stops)	km/h	34.9	24.2
Average speed (excluding stops)	km/h	38.8	30.4
Maximum acceleration	m/s^2^	3.08	3.09
Maximum deceleration	m/s^2^	5.05	3.37
Urban part va_pos95_ *	m^2^/s^3^	11.68	12.75
Urban part RPA **	m/s^2^	0.211	0.246

* va_pos95_—95th percentile of positive acceleration and speed; ** RPA—Relative Positive Acceleration.

**Table 6:** Specifications of the HORIBA OBS-2200 PEMS system.

Analyzer	Measurement Range	Measured Component	Accuracy
NDIR	0–20 vol%	CO_2_	± 2.5%
NDIR	0–10 vol%	CO	
CLD	0–3000 ppm	NO_X_	
FID	0–10,000 ppm	HC	
Pitot tube (exhaust mass flow)	0–65 m^3^/min	exhaust flow rate	±1.5% of full scale or ±2.5% of reading (whichever is greater)

**Table 7:** Hyperparameter settings and structural characteristics of bagged-tree regression models developed for exhaust emission prediction.

Parameter	*V_a_CO*_2_	*V_a_P_rl__CO*_2_	*V_a_CO*	*V_a_P_rl__CO*	*V_a_CH*_4_	*V_a_P_rl__CH*_4_	*V_a_HC*	*V_a_P_rl__HC*	*V_a_NO_X_*	*V_a_P_rl__NO_X_*
Number of trees	30	30	30	30	30	30	30	30	30	30
Minimum leaf size	8	8	8	8	8	8	8	8	8	8
Number of splits	1412	1395	1277	1217	1297	1300	1224	1215	1208	1193
Maximum tree depth	32	30	24	23	23	24	28	26	23	25

Common model settings for all variants: Split criterion = Mean Squared Error (MSE); Ensemble method = Bootstrap Aggregation (Bagging); Input feature normalization = Not applied. Table note: All regression models were implemented in MATLAB R2025b using the Regression Learner app.

**Table 8:** Results of emission model validation under real-world driving conditions. Summary of accuracy metrics for models based on vehicle speed and acceleration (*V_a*) and models extended with road load power (*V_a_P_rl_*), representing an additional parameter describing the actual vehicle load.

Model	r	R^2^	RMSE [g/s]	MAE [g/s]	MAPE [%]
*V_a_CO*_2_	0.57959	0.33593	0.95191	1.33721	0.86927
*V_a_P_rl__CO*_2_	0.56765	0.32222	0.99050	1.41561	0.83879
*V_a_HC*	0.03286	0.00107	0.00793	0.03460	1.23045
*V_a_P_rl__HC*	0.08756	0.00766	0.00808	0.03420	1.24040
*V_a_NO_X_*	0.32782	0.10747	0.01984	0.03144	5.88348
*V_a_P_rl__NO_X_*	0.32002	0.10241	0.02164	0.03543	6.10733
*V_a_CO*	0.07066	0.00499	0.01004	0.02064	2.23744
*V_a_P_rl__CO*	0.07525	0.00566	0.01067	0.02178	2.53136
*V_a_CH*_4_ *	0.03047	0.00092	0.00523	0.02517	0.87421
*V_a_P_rl__CH*_4_ *	0.03962	0.00157	0.00527	0.02476	0.88909

* Methane emissions estimated based on the percentage share of CH_4_ in total hydrocarbon (HC) emissions determined from laboratory tests.

## Data Availability

The original contributions presented in this study are included in the article. Further inquiries can be directed to the corresponding author.

## Abbreviations

The following abbreviations are used in this manuscript:

*a*	Vehicle acceleration
aDyno	Acceleration on dynamometer
*A_d_*	Frontal area of the vehicle
CLD	Chemiluminescence detection
CH_4_	Methane
CH_4_*	Methane emissions estimated based on the percentage share of CH_4_ in total hydrocarbon (HC) emissions determined from laboratory tests
*c_gas_*	Measured concentration of the gaseous component
C_CO_2__	CO_2_ concentration in the diluted exhaust gases
C_HC_	HC concentration in the diluted exhaust gases
C_CO_	CO concentration in the diluted exhaust gases
C_d_	Volume concentration of gaseous component in the dilution air
*c_d_*	Aerodynamic drag coefficient in the longitudinal direction
*C_e_*	Measured volume concentration of gaseous component in the diluted exhaust gases
*C_i_*	Volume concentration of gaseous component in the dilution air
CNG	Compressed Natural Gas
CO	Carbon monoxide
CO_2_	Carbon dioxide
CVS	Constant Volume Sampling
DF	Dilution factor
EC	Energy consumption
*egas*	Average emission of the exhaust gas component
*eCO*	Average emission of carbon monoxide
*eCO*_2_	Average emission of carbon dioxide
*eCH*_4_	Average emission of methane
*eHC*	Average emission of total hydrocarbons
*eNO_X_*	Average emission of nitrogen oxides
*δ*	Rotational mass coefficient
*η*	Drivetrain efficiency coefficient
*f*_0_	Constant road load coefficient
*f*_1_	First-order road load coefficient
*f*_2_	Second-order road load coefficient
*f_t_*	Rolling resistance coefficient
*φ*	Load coefficient dependent on the vehicle category
*e_gas_*	Average gaseous exhaust emission [g/km]
FID	Flame ionization detector
F_rl_	Road load force
*g*	Gravitational acceleration
H	Vehicle height
HC	Total hydrocarbons
HHV	Higher heating value
IRD	Infrared detector
IVE	International vehicle emissions
LHV	Lower heating value
LNG	Liquefied natural gas
*m_gas_*	Mass emission rate of the exhaust gas component
*m_CO_*	Mass emission rate of carbon monoxide
*m_CO_*__2__	Mass emission rate of carbon dioxide
*m_CH_*__4__	Mass emission rate of methane
*m_HC_*	Mass emission rate of total hydrocarbons
*m_NO_X__*	Mass emission rate of nitrogen oxides
ML	Machine Learning
*m_l_*	Gross vehicle weight rating
*m_o_*	Additional equipment mass
*m_t_*	Test vehicle mass
*m_R_*	Actual vehicle mass
*m_r_*	Test vehicle mass in on-road tests
*m_ro_*	Vehicle mass in running order
*m_vl_*	Maximum vehicle load
MON	Motor octane number
MPI	Multi-point injection
N_2_	Nitrogen
N_2_O	Nitrous oxide
NO	Nitric oxide
NDIR	Non-dispersive infrared
NEDC	New European Driving Cycle
NH_3_	Ammonia
NO_X_	Nitrogen oxides
OBD	On-board diagnostics system
O_2_	Oxygen
*P_rl_*	Road load power
*P_rl_Dyno*	Dynamometer road load power
PEMS	Portable Emissions Measurement Systems
*r*	Pearson correlation coefficient
RDE	Real driving emissions
RON	Research octane number
RMSE	Root Mean Square Error
MAE	Mean absolute error
MAPE	Mean absolute percentage error
*ρ_gas_*	Density of the compound
*ρ_CO_*	Density of the CO
*ρ_CO_*__2__	Density of the CO_2_
*ρ_NO_X__*	Density of the NO_X_
*ρ_HC_*	Density of the HC
*ρ_NG_*	Density of the natural gas
*q_mew_*	Measured exhaust gas flow rate
RPA	Relative Positive Acceleration
SI	Spark-ignition
TWC	Three-way catalytic converter
UDC	Urban Driving Cycle
V	Vehicle speed
*VDyno*	Simulated vehicle speed on a chassis dynamometer
VSP	Vehicle specific power
*va_pos_*_95_	95th percentile of positive acceleration and speed
*W*	Vehicle width
*w*	road grade
WLTC	Worldwide Harmonized Light Vehicles Test Cycle
